# Determination of antimicrobial resistance patterns of *Escherichia coli* isolates from farm workers in broiler poultry production and assessment of antibiotic resistance awareness levels among poultry farmers in Lusaka, Zambia

**DOI:** 10.3389/fpubh.2022.998860

**Published:** 2023-01-10

**Authors:** Mwaba Mwansa, Mercy Mukuma, Esther Mulilo, Geoffrey Kwenda, Geoffrey Mainda, Kaunda Yamba, Flavien Nsoni Bumbangi, Elizabeth Muligisa-Muonga, Nelson Phiri, Isaac Silwamba, John Bwalya Muma

**Affiliations:** ^1^Department of Basic Medical Sciences, Michael Chilufya Sata School of Medicine, Copperbelt University, Ndola, Zambia; ^2^Department of Food Science and Nutrition, School of Agricultural Sciences, University of Zambia, Lusaka, Zambia; ^3^Department of Biomedical Sciences, School of Health Sciences, University of Zambia, Lusaka, Zambia; ^4^Department of Veterinary Services, Public Health Unit, Ministry of Fisheries and Livestock, Lusaka, Zambia; ^5^Department of Pathology and Microbiology, University Teaching Hospitals, Lusaka, Zambia; ^6^Department of Medicine and Clinical Studies, School of Medicine, Eden University, Lusaka, Zambia; ^7^Department of Environmental Health, School of Medicine, Eden University, Lusaka, Zambia; ^8^Livestock Services Cooperative Society, Department of Laboratory and Diagnostics, Lusaka, Zambia; ^9^Department of Disease Control, School of Veterinary Medicine, University of Zambia, Lusaka, Zambia

**Keywords:** antimicrobial resistance, poultry, ESBL *E. coli*, poultry farmworker, Zambia

## Abstract

The challenges posed by antibiotic-resistant pathogens have continued to increase worldwide, particularly in resource-limited countries. Human-livestock interactions are implicated in the complex AMR causal web. A cross-sectional study was conducted in four districts of Lusaka Province, Zambia to determine the antibiotic resistance patterns, ESBL production of *E. coli* isolated from stool samples of broiler poultry farm workers, and to assess poultry farmers' antibiotic resistance awareness. Sixty-six human stool samples were collected and processed for *E. coli* isolation, antibiotic resistance testing, and screened for ESBL production. In addition, 80 farmers were assessed for their level of awareness on antibiotic resistance. A total of 58 single *E. coli* isolates were obtained which showed high (87.9%) resistance to tetracycline, trimethoprim/sulfamethoxazole (48.3%), and ampicillin (46.8%); followed by nalidixic acid (19.0%), ciprofloxacin (12.1%), cefotaxime (8.6%) and chloramphenicol (5.2%). The prevalence of AMR *E. coli* was 67.2%, and 29.3% were MDR. Two (3.4%) isolates were identified to be ESBL producers, harboring the *CTX-M* gene. The study results also showed that broiler farmers were aware and knowledgeable of antibiotic resistance, although knowledge about its impact on human health was low. This study demonstrated the presence of resistant and ESBL producing *E. coli* among poultry farm workers.

## 1. Introduction

Poultry and poultry products provide a significant and cheap source of protein. As a result, consumer demand has increased worldwide, and the total global production is projected to increase by 3% annually to offset this demand (1, 2). In Zambia, poultry and poultry products are important sources of food and income in most households (3). The poultry industry is the largest contributor to the country's livestock gross domestic product estimated at 48% (4) largely comprising of indigenous, layers and broiler chickens (5). Since 2000, the poultry industry has been growing steadily at a growth rate of between 8 and 10%, attributed to population growth which stands at 2.8% per year (6), increased disposable income, emerging middle class and rapid urbanization which drives the demand for poultry and poultry products due to very competitive prices comparable to other sources of animal proteins such as beef, lamb, pork and fish (5).

Poultry industry in Zambia encompasses both small and commercial scale poultry farmers, with former dominating the industry (4). The specific number of people rearing broiler chickens is unknown (4). Most poultry farmers are mostly settled along the line of rail stretching from Livingstone through Lusaka to Kitwe as well as Chililabombwe on the border with Katanga Region in the Democratic Republic of Congo (4). In 2018, the Ministry of Fisheries and Livestock estimated that there were 6.8 million broiler chickens, 1.7 million layers and 15.4 million village chickens, with Lusaka, Copperbelt, and Central provinces producing nearly 75% of the total broiler birds in Zambia, as of 2018 (5).

For small-scale farmers, poultry is usually kept in the backyard of the farmers' houses under intense conditions such as high population densities, promoting profitability (3). Knowledge about poultry production is usually acquired from friends already in the business, leading to several management dilemmas such as hygiene, feed quality, treatment, and control of infections (7).

Failure in poultry management and biosecurity contribute to the inappropriate use of a wide range of antimicrobials for prophylactic, therapeutic, and growth-promotion purposes over a prolonged period (8). This inappropriate use through overuse and misuse of antimicrobials promotes the selective pressure of antimicrobial-resistant (AMR) microorganisms and may promote the emergence and spread of AMR strains (8–10). Antibiotic use in the Zambian poultry industry is prevalent (5) and studies on broiler chickens have reported AMR strains of microorganisms (11–13). These resistant strains can be transmitted to humans through the environment, food products, and direct contact with food animals (1, 14, 15). Further, resistant strains of commensal microorganisms have a potential to pass resistance genes to human pathogens which in turn can cause health complications in human populations (16). Farm workers are constantly exposed to poultry and are at of risk acquiring AMR microorganisms.

In this study, *Escherichia coli* (*E. coli*) was of particular interest because it is a critical specie that can be used as an indicator microorganism for AMR in a particular host or environment, including multidrug resistance (MDR) - resistance to three or more antimicrobial classes (17).

Antimicrobial resistance trends in *E. coli* are of particular concern given their ubiquity in the environment and human and animal hosts, and the relative ease with which they acquire and transmit genetic determinants that confer resistance to most classes of antibiotics (18).

Transmission of resistant *E. coli* strains from poultry to humans is of great concern because the acquired resistance can occur in pathogenic bacteria and the endogenous bacterial flora of exposed individuals or populations (19, 20). AMR poses challenges in Diering and Mitchell (16) managing and treating infections in both food animals and humans (21, 22) due to the increase in number of infections, prolonged duration of infection, limited choice of empirical treatment antibiotics, and complications resulting from failed treatment; consequently result in increased cost to society.

*E. coli* can be resistant to several different classes of antibiotics. However, β-lactamase production is the most critical mediation of resistance to broad-spectrum β-lactams (23). β-lactamases are a broad class of enzymes that are often encoded on plasmids. They confer resistance to penicillins and cephalosporins and are an emerging cause of multidrug resistance in *E. coli*. Extended-spectrum β-lactamase (ESBL)-producing *E. coli* has been documented both in humans and poultry, and the prevalence has increased significantly over the past decade (24).

Resistance to newer drugs and the emergence of resistance to β-lactam antibiotics have been reported in *E. coli* and other *Enterobacteriaceae* from extraintestinal and environmental sources in Zambia (25, 26).

Regardless of this evidence of AMR in Zambia, no such study has been conducted on poultry farmworkers. In addition, knowledge on antibiotic use, and the impact of antibiotic resistance in broiler chickens on human health among broiler farmers is unknown. Improving knowledge about AMR among poultry farmers is particularly important in low-income countries, where diarrhoeal diseases are highest (27). A study by Tebug et al. found that the awareness of AMR and its adverse effects on human health in food animal production, particularly poultry production, ranged from moderate to low among animal health professionals in 20 sub-Saharan African countries (28).

There is a paucity of information on the role of poultry farmworkers in the epidemiology of resistant pathogens from poultry to humans. Therefore, this study aimed to determine the AMR patterns of *E. coli* isolated from stool samples of farmworkers and assess broiler farmers' awareness of antibiotic resistance and the knowledge about its impact on human health in selected districts in Lusaka Province, Zambia.

## 2. Materials and methods

### 2.1. Study area and design

This cross-sectional study was conducted in four districts of Lusaka Province (Chilanga, Chongwe, Kafue, and Lusaka) between August 2017 and December 2018, districts in the province with high populations of poultry businesses. Lusaka Province was also selected because it is the home to the capital city and a trading hub for poultry and poultry products.

### 2.2. Sample size and sampling method

The sample size was determined using the formula for an unknown population.

The formula used to determine the target sample size was as follows:


$$N={\left(Z\right)}^{2*}\;S^{*}(1-S)/{\left(E\right)}^{2}$$

Where: *N* = required minimum sample size; The *Z*-Standard value (1.96) corresponds to a confidence level at 95%; *S* = sample standard deviation at 0.5; *E* = accepted magnitude of error at 0.05.

The minimum target sample size was estimated at 384 participating broiler farmers, adjusted at 6% attrition for non-consent bias and inability to locate the broiler farmers to get 407.

There was no information on the number of farmers who reared broiler chickens. Most broiler farmers were mostly seasonal farmers. According to Phiri et al. a seasonal farmer was defined as the farmer who keeps broiler chicken when the production parameters including the cost of feed, and cost of medicines are favorable and stops when they are not (11). The scaling of the farmers was done according to the National Surveillance Protocol for AMR in broiler chickens currently being applied by Government as follows: small scale (< 500 birds per poultry house); medium scale (500–1,000 birds per poultry house); commercial (>1,000 birds per poultry house). Therefore, broiler poultry farmers were identified and located using the snowball sampling technique. Farmers in production were initially identified with the help of a local veterinary assistant or livestock officer. Such farmers would then lead to other farmers in the season of production.

Firstly, purposive sampling was employed, including only broiler farmers. As the population of broiler farmers was unknown and with the limitation of locating them, the maximum sample of 89 participants (farms located) was attained for this study from all the selected districts. Eighty farmers responded to the questionnaire: Chilanga (*n* = 21), Chongwe (*n* = 15), Kafue (*n* = 20), and Lusaka (*n* = 24) and 66 participants (farm workers) consented to provide stool samples Chilanga (*n* = 13), Chongwe (*n* = 13), Kafue (*n* = 13), and Lusaka (*n* = 27).

Farmers, from small farms without farmworkers, who performed the daily work of caring for the broiler chickens responded to the questionnaire and provided the stool sample. We sampled concerting farmers and farmworkers who had routine contacts with chickens.

At least one person from each farm was sampled among those who actively worked daily with broiler chickens in the poultry houses.

Participants were given a sterile stool container into which to place the early morning stool sample. The samples were collected and transported to the University of Zambia, Public Health Laboratory, and analyzed within 7 h of receiving them. In addition, a questionnaire was administered to the poultry farmer or his/her proxy to obtain poultry farm demographic data, poultry farm characteristics, and management, and to assess awareness and knowledge about antibiotic resistance.

### 2.3. Assessment of antibiotic resistance awareness levels

Data was collected through face-to-face interviews using a semi-structured questionnaire to assess broiler farmers' awareness and basic knowledge about antibiotic resistance and its impact on human health. The questionnaire included the following sections: farm identification, demographic characteristics, general poultry farm characteristics, antibiotic use, and awareness and knowledge about antibiotic resistance and its impact on human health, as well as the source of information on antibiotic resistance.

Awareness was defined as having an understanding/hearing of antibiotic resistance and respondents were expected to say yes if they knew or had heard about antibiotics resistance. Knowledge about antibiotic resistance was defined as having the correct understanding of or facts on antibiotic resistance. The responses on what antibiotic resistance was, were used to determine basic knowledge about antibiotic resistance.

The questionnaire was pretested among 15 non-participating broiler poultry farmers in Chisamba District, Central Province, with similar characteristics. The findings after pretesting the questionnaire allowed for a modification to ensure the clarity and reliability of the questionnaire.

### 2.4. Laboratory analysis

#### 2.4.1. Isolation of *E. coli*

Upon receipt at the laboratory, samples were pre-enriched in Buffered Peptone Water (BPW) broth (Oxoid Ltd., Basingstoke, Hampshire, UK) by adding 1 g of sample to 9 ml of BPW using a sterile orange stick and incubating overnight at 37°C. A loopful of the sample was then cultured on MacConkey agar (Oxoid Ltd., Basingstoke, Hampshire, UK) using the streaking method and incubated overnight at 37°C. After incubation, small pink colonies were stained using Gram's staining technique and examined under a light microscope. Colonies showing Gram-negative short rods were then sub-cultured onto Eosin Methylene Blue (EMB) agar (Oxoid Ltd, Basingstoke, Hampshire, UK). Colonies showing a metallic green sheen were sub-cultured onto nutrient agar (Oxoid Ltd, Basingstoke, Hampshire, UK) and incubated overnight at 37°C before being subjected to identification tests.

#### 2.4.2. Biochemical identification of *E. coli* isolates

Pure cultures of suspected *E. coli* isolates on nutrient agar were first subjected to biochemical testing for identification. Biochemical tests performed included Triple Sugar Iron (TSI), Urease, Citrate and Sulfide, Indole, Motility (SIM). Briefly, one to five single pure colonies of bacteria were picked using a sterile inoculating needle and stubbed into the medium and then streaked on the surface the of TSI agar slants. The tubes were then incubated at 37°C for 18–24 h with loosened caps and results recorded. Change in color of the butt and slant from red to yellow was noted, as well as presence or absence of gas and hydrogen sulfide. Materials from the same pure colonies of bacteria used for TSI test were later used for all the other biochemical tests. Sterile inoculating needle was used for each subsequent test. Bacteria was lightly stubbed into the center of the Urea agar slants. The Urea agar slant tubes were then incubated at 37°C for 18–24 h with loosened caps and results recorded. Change in color of the media from yellow to pink/red was noted as a positive result while no change in color of the media was noted as a negative result. Bacteria was lightly stubbed into the Citrate agar slant ~1 cm from the bottom of the tube and made sure not to twist the needle. The tubes were then incubated at 37°C for 18–24 h with loosened caps and results recorded. Change in color of the media from green to blue was noted as a positive result while no change in color of the media was noted as a negative result.

A single stab of bacteria was made into the tube of SIM agar ~1 cm from the bottom of the tube and made sure not to twist the needle. The tubes were then incubated at 37°C for 18–24 h with loosened caps and results recorded. Motility and presence or absence of hydrogen sulfide were noted. Three drops of Kovacs reagent were then applied to the surface of the media, and the development of a pink to red color was noted.

Biochemical identification was according to developed standard operating procedures (SOPs) (29, 30), technically supported by the reviewers from the WHO-Advisory Group on Integrated Surveillance of Antimicrobial Resistance (AGISAR).

#### 2.4.3. Molecular identification of the *E. coli* isolates

To confirm the identity of the *E. coli* isolates after biochemical testing, the isolates were subjected to 16S rRNA sequencing according to the method described by Weisburg et al. (31). Briefly, a loopful of bacterial colonies from the nutrient agar plates were transferred into a sterile Eppendorf tube containing 100 μL of nuclease-free water. The cell suspension was mixed by vortexing and placed on a heating block at 80°C for 10 min to allow cells' thermolysis. The lysate was then centrifuged at 6,000 rpm for 2 min at 4°C, and the supernatant was collected into new sterile Eppendorf tubes. The supernatant samples were then used for PCR amplification and 16S rRNA sequencing to confirm the isolates.

#### 2.4.4. Determination of drug susceptibility patterns of *E. coli* isolates

The Kirby-Bauer disk diffusion test was performed on Müeller-Hinton agar (Oxoid Ltd, Basingstoke, Hampshire, UK) according to the Clinical and Laboratory Standards Institute (CLSI) guidelines (32). The drugs tested included amoxicillin-clavulanic acid (30 μg), ampicillin (10 μg), cefotaxime (30 μg), chloramphenicol (30 μg), ciprofloxacin (5 μg), colistin sulfate (10 μg), imipenem (10 μg), nalidixic acid (30 μg), trimethoprim/sulphamethoxazole (25 μg), and tetracycline (30 μg). Plates were prepared as follows: A well-isolated colony was selected from an overnight culture on Nutrient agar (Oxoid Ltd., Basingstoke, Hampshire, UK) with a sterile swab and suspended in 5 ml of sterile normal saline. The turbidity of the suspension was adjusted with sterile saline to obtain a suspension equivalent to 0.5 McFarland standard and evenly spread on the Müeller-Hinton agar (Oxoid Ltd., Basingstoke, Hampshire, UK). An automatic dispenser (Oxoid Ltd., Basingstoke, Hampshire, UK) was then employed to apply the antibiotic discs onto the plates and incubated at 37°C for 16–18 h. Zones of inhibition were measured with a digital vernier caliper, and endpoints were determined based on the areas showing no bacterial growth visible to the naked eye. Results were interpreted according to the CLSI guidelines (32). *E. coli* control strain ATCC 25922 was used as a control strain for the test. A multidrug-resistant (MDR) strain was defined as resistant to at least three different classes of antimicrobial agents.

#### 2.4.5. Determination of ESBL-producing *E. coli* isolates

The *E. coli* isolates were also screened for ESBL production using the cephalosporin/clavulanate combination disc method. The isolates were first tested against cefpodoxime and cefotaxime (Mast Diagnostics Ltd., Merseyside, U.K.) as an indicator of cephalosporins and ESBL production was confirmed phenotypically using a combination of discs (cefpodoxime/clavulanic acid cefotaxime/clavulanic acid) (Mast Diagnostics Ltd., Merseyside, U.K.) according to CLSI guidelines (32). The test was considered positive if the inhibition zone diameter was ≥5 mm larger with clavulanic acid than without.

The ESBL-positive isolates were further screened for the presence of common ESBL genes, *CTX-M, TEM*, and *SHV*, by PCR using the following primers *CTX-M* TCTTCCAGAATAAGGAATCCC (Forward), CCGTTTCCGCTATTACAAAC (Reverse), *TEM* TCCGCTCATGAGACAATAACC (Forward), TTGGTCTGACAGTTACCAATGC (Reverse), and *SHV* TGGTTATGCGTTATATTCGCC (Forward), GGTTAGCGTTGCCAGTGCT (Reverse).

### 2.5. Data analysis of antibiotic resistance awareness and AMR in *E. coli*

The data collected through questionnaires was entered in Excel Spreadsheets and cleaned, then exported to SPSS version 22 for analysis. Descriptive statistics were used to analyze all the variables of interest, including age, gender, education level, marital status, awareness, and knowledge about antibiotic resistance, knowledge about the impact of antibiotic resistance on human health and sources of information on antibiotic resistance. Percentages were then generated. The level of confidence was set a 95% (α = 0.05), and Pearson Chi-Square was used to measure the association between categorical variables. Variables included both independent (age, gender, marital status, education, and source of information) and dependent variables (awareness and knowledge about antibiotic resistance).

The proportion of AMR isolates of *E. coli* was calculated by dividing the number of AMR *E. coli* over the total isolates. Antimicrobial susceptibility test results were entered into and analyzed in WHONET 2018. Percentage resistance and susceptibility of the *E. coli* isolates to different antibiotics were calculated. The proportion of ESBL-producing *E. coli* was determined, and the encoding genes were profiled.

### 2.6. Ethical considerations

Permission to conduct the study was sought from the Lusaka Provincial Health Office. Before specimen collection, an informed consent statement was obtained from all study participants. Participants were free to accept or decline to participate in the study. No incentive was provided for consenting to participate in the study. To maintain confidentiality, study participants were allocated study-specific codes, and the study data was kept confidential. Ethics clearance was sought from ERES Converge IRB (IRB No. 00005948) Ethics Committee (Study Reference: 2017-Aug-003).

## 3. Results

In this study, 89 broiler farmers from four selected districts of Lusaka province were enrolled (Table 1). Out of this, total of 80 broiler farmers (90%) responded to the questionnaire on antibiotic resistance awareness levels and 66 stool samples were collected from farmworkers (74.2%) who were in constant contact with the birds (Table 1).

**Table 1:** Distribution of farmers, samples, *E. coli* isolates and ESBL producing *E. coli*.

**District**	**Farmers**	**Responses to questionnaire**	**Stool samples**	***E. coli* isolates (% yield)**	***E. coli*** **resistance (AMR)**		**ESBL producing** ***E. coli***	

					**At least one antibiotic (%)**	≥**3 antibiotics (MDR) (%)**	**Isolates**	**Detectable** ***CTX-M*** **gene**
Chilanga	23 (26%)	21 (91.3)	13 (56.5)	12 (92.3)	6 (50.0)	3 (25.0)	1 (8.3)	0 (0.0)
Chongwe	17 (19%)	15 (88.2)	13 (76.5)	12 (92.3)	9 (75.0)	4 (33.3)	1 (8.3)	1 (8.3)
Kafue	22 (25%)	20 (90.9)	13 (59.1)	11 (84.6)	9 (81.8)	2 (18.2)	0 (0.0)	0 (0.0)
Lusaka	27 (30%)	24 (88.9)	27 (100)	23 (85.2)	15 (65.7)	8 (34.8)	3 (13.0)	1 (8.3)
Total	89 (100%)	80 (89.9)	66 (74.2)	58 (87.9)	39 (67.2)	17 (29.3)	5 (8.6)	2 (3.4)

### 3.1. Antibiotic resistance awareness

Most broiler farmers were small-scale (97.6%), of which 56.3% were females, and 41.3% were males, rearing < 500 birds per cycle in backyard poultry houses (72%). Only one female and one male farmer belonged to medium-scale and commercial-scale broiler production categories, respectively (Table 2). The majority of the respondents were in the 21–30 years age category (38.8%), with the 51–60 years age category being the lowest (3.8%) (Table 3). The results also showed that most broiler farmers were married (57.5%) and 36.3% were single. The majority (32.5%) had attained senior secondary school education (Table 3).

**Table 2:** Poultry farm characteristics and antibiotics use (*N* = 80).

**Poultry farm characteristics**	**Categories**	**Percentage (%)**
Farm category	Small scale	97.6
	Medium scale	1.2
	Commercial	1.2
Number of birds per house	< 500	97.5
	>500	2.5
Reason for rearing	Meat for sale	1.2
Broiler chickens	Meat for sale plus consumption	98.8
Do you use antibiotics to treat sick birds?	Yes	69
	No	31
Antibiotics used?	Oxytetracycline	90
	Doxycycline	90
	Sulfamethoxazole	10
	Gentamycin	10
Are antibiotics used for growth promotion?	Yes	35
	No	65
How do you sell the birds when ready for market?	Live	47.5
	Both live and dressed	52.5
Is poultry house in the backyard?	Yes	90
	No	10

**Table 3:** Broiler farmers' social demographic characteristics and awareness level on AMR (*N* = 80).

**Socio-demographic characteristics**		**Proportion of respondents (%)**	***P*-value awareness**	**Awareness of**	
				**antibiotic resistance (%)**	

				**Yes**	**No**
Age (yrs.)	18–20	5	0.166	0	0
	21–30	38.8		48.4	51.6
	31–40	26.3		61.9	38.1
	41–50	13.8		72.7	27.3
	51–60	3.8		33.3	67.7
	>60	12.5		60	40
Marital status	Married	57.5	0.447	56.5	43.5
	Single	36.3		44.8	55.2
	Divorced	1.3		0	100
	Widowed	5		100	0
Education level	None	7.5	0.36	50	50
	Primary	7.5		16.7	83.3
	Junior secondary	22.3		50	50
	Senior secondary	32.5		57.7	42.3
	Tertiary	30		62.5	37.5
Gender	Female	57.5	0.289	59.1	40.9
	Male	42.5		47	53

Most (98.8%) of broiler farmers reared broiler chickens for sale and home consumption. Majority of farmers (68.8%) used antibiotics to treat sick birds. Commonly used antibiotics were oxytetracycline, doxycycline, sulfamethoxazole, and gentamycin (Table 2). Thirty-five percent (35%) used antibiotics for the growth promotion of their birds. The results also showed that most farmers (52.5%) sold their chickens both live and dressed, with 47.5% of them selling only live chickens (Table 2).

The results showed no significant (*p* > 0.05) association between awareness of antibiotic resistance and socio-demographic characteristics (Table 3). However, results showed a considerable difference in age, gender, and marital status regarding awareness.

With regard to marital status, the results show that the widowed (100%) were the most aware of the antibiotic resistance, while the divorced were least. In terms of education levels, farmers who had reached tertiary education (62.5%) were the most aware of antibiotic resistance. In contrast, the least aware (16.7%) were those who only attained primary education. In terms of gender, women (59.1%) were relatively more aware of antibiotic resistance than their male counterparts (47%).

The results also showed that the majority (67%) of broiler farmers had a basic understanding of antibiotic resistance (Table 4). However, majority (74%) of the broiler farmers were unaware of the impact of antibiotic resistance on human health (Table 4). Some of the correct responses from the interviews on antibiotic resistance included “overuse of antibiotics that make them stop working thus cannot treat infections,” “bacteria don't respond to drugs administered,” and “disease does not go away after taking antibiotics.” The incorrect responses included “something about chlorine,” “drug reactions,” “cholera prevention,” and “can't explain.”

**Table 4:** Knowledge of antibiotic resistance and its impact on human health (*N* = 80).

**AMR knowledge and its impact on human health**	**Response**	**Percentage (%)**
Are you aware of antibiotic resistance in broiler production?	Yes	54
	No	46
If you are aware, what is antibiotic resistance?	Failure of antibiotics treating infections	67
	Do not know	33
What is the impact of antibiotic resistance to human health?	Persistent infection	26
	Do not know	74
Broiler farmers who were aware and knowledgeable about antibiotic resistance in broiler chickens and its impact on human health		14

The primary sources of information on antibiotic resistance among the broiler farmers were the suppliers of broiler chicks (70.1%) and peers/friends (16.1%). Others were television (5.8%), hospital (4.6%), and social media (3.5%).

### 3.2. AMR *E. coli*

A total of 66 sample stools were collected and 58 *E. coli* were isolated. Thirty-nine (67.2%) *E. coli* isolates showed resistance to at least one antibiotic and 17 (29.3%) to at least three antibiotics (Table 1).

Five *E. coli* isolates were found to be ESBL-producers using the phenotypic testing approach, but only two of the isolates had a detectable ESBL gene (*CTX-M*). No *TEM* or *SHV* gene was detected in any of the isolates tested (Table 1).

*E. coli* isolates exhibited the highest resistance to tetracycline (61.7%, *n* = 36), sulfamethoxazole/trimethoprim (48.3%, *n* = 28), and ampicillin (46.8%, *n* = 27) (Table 5). One hundred percent (100%) susceptibility was observed to colistin and imipenem (Table 5).

**Table 5:** Antibiotic resistance profiles for *E. coli* isolates.

**Antibiotic name**	**Number**	**Breakpoints**	**%*R***	**%*I***	**%*S***	**%*R* 95 %C.I**.
Amoxicillin/clavulanic acid	58	14–17	0.0	5.2	94.8	0.0–7.7
Ampicillin	58	14–16	46.8	2.1	51.1	32.4–61.8
Chloramphenicol	58	13–17	5.2	5.2	89.7	1.4–15.3
Ciprofloxacin	58	16–20	12.1	0.0	87.9	5.4–23.9
Colistin	58	S ≥ 11	0.0	0.0	100.0	0.0–7.7
Cefotaxime	58	23–25	8.6	0.0	91.4	3.2–19.7
Imipenem	58	20–22	0.0	0.0	100.0	0.0–7.7
Nalidixic acid	58	14–18	19.0	5.2	75.9	10.3–31.9
Trimethoprim/sulfamethoxazole	58	11–15	48.3	1.7	50.0	35.2–61.7
Tetracycline	58	12–14	61.7	0.0	38.3	46.4–75.1

## 4. Discussion

This study aimed to assess poultry farmer awareness and basic understanding of antibiotic resistance and determine the AMR patterns of *E. coli* isolated from stool samples of poultry farm workers.

Lusaka district had the highest number of broiler farmers investigated. These results are consistent with the findings of Lungu which showed that most of the broiler production in Zambian occurred within Lusaka Province (4). Lusaka district is a densely populated area with a high broiler consumption rate. Therefore, many people engage in broiler poultry production as a source of income and to meet the demand for chicken meat for the city's growing population.

Majority of the broiler farmers were small-scale (97.6%). This high number of small-scale farmers could be attributed to the high cost and inadequate capital required to operate at commercial production (33).

This study also found that for most farmers in this study, the birds were reared in backyard poultry houses usually with high bird stocking density and poor ventilation. Backyard poultry production is often associated with low levels of biosecurity (32), which increases the risk of disease occurrence and consequent use of antibiotics (34). Moreover, backyard poultry production contributes to the emergence of AMR due to the increased need and extended use of antibiotics for growth promotion, disease prevention, and infection treatment (35, 36). Hedman et al. and Hoelzer et al. who reviewed antimicrobial drug use and antimicrobial resistance in poultry production found that in low-resource settings backyard poultry production contributes to the emergence of AMR due to the shortcomings in biosafety management that is commonly addressed with increased use of antimicrobial agents (35, 36).

Most broiler chickens are sold both live and dressed, reflecting consumers' preference when buying broiler chickens. Joshua Olorunwa's study also showed that most broiler farmers reared broiler chickens for meat and home consumption, with only a few rearing broilers for sale (37). Broiler chickens traded on the market were either processed at a recognized slaughter facility or at the home of the farmer, particularly done by back yard farmers. Chickens processed at home posed a relatively higher risk of carrying AMR pathogens than those processed in a slaughter facility because of increased likelihood of cross-contamination resulting from unhygienic processing conditions (11, 12, 34). The cross contamination and spread of AMR microorganisms to the environment and the public, in general, could also be due to the marketing of live birds at local open markets and roadside stalls, with limited sanitation, where humans and food animals interface (38–41). With these different activities during broiler poultry production, there could be a transmission of resistant microorganisms from poultry to the farmworkers and vice versa due to frequent interactions.

This study showed no association between awareness of antibiotic resistance and socio-demographic characteristics such as age, marital status, education, and gender which could be attributed to the small sample size. Perhaps other factors such as broiler farming experience may influence awareness of antibiotic resistance among the study participants.

Even though statistical results showed no association between the characteristics mentioned earlier and antibiotic resistance awareness, results showed a considerable difference in age, gender, and marital status regarding awareness. For instance, most farmers were aware of antibiotic resistance, particularly from the 41–50 age category. This is probably because most of these, had adequate levels of experience to understand these effects. Unsurprisingly the age group of 15–20 years was utterly unaware of antibiotic resistance. Most individuals in this category were beginning their broiler chicken business and did not have much experience. A study by Pham-Duc and Sriparamananthan found females to have better knowledge of antibiotics and AMR in comparison to males (42). This was attributed to females being more likely to seek help and visit healthcare facilities, thus receiving more information on antibiotics (42). Concerning marital status, the widowed were more aware, which could be attributed to age and adequate levels of experience and knowledge in poultry production. In addition, broiler production could be their main or only source of income, thus putting effort to acquire knowledge in production.

Knowledge about the impact of antibiotic resistance on human health was low. This lack of knowledge could be partly attributed to inadequate formal sensitization of antibiotic resistance's impact on human health. These results agree with Katakweba et al. (42, 43) in Tanzania, who also observed an extreme lack of awareness of health risks associated with AMR among food animal producers, posing a serious human health risk. Insufficient knowledge about the impact of AMR on human health is of great concern, as it can adversely affect human health, especially if special care is not taken into account to eradicate or minimize antibiotic resistance. Most broiler farmers obtained information from the chick sellers they regularly contacted. Other sources of information included hospitals, peers, social media, and television. There is a need to identify effective channels of communication to increase the dissemination of AMR information and its possible risks to human health.

The prevalence of AMR and MDR *E. coli* was found to be 67.2 and 29.3%, respectively. This prevalence was higher compared to 39.7% observed by Aworh et al. (44) who carried out a similar study in Abuja, Nigeria (44). Resistance to tetracycline (61.7%), sulfamethoxazole/trimethoprim (48.3%), and ampicillin (46.8) was highest. Aworh et al. also obtained similar results of resistance; 83.3, 79.2, and 77.1% to tetracycline, sulphamethoxazole/trimethoprim, and ampicillin, respectively. A recent study on AMR in young children in Lusaka and Ndola districts of Zambia showed that *E. coli* had highest resistance against ampicillin (78.0%), trimethoprim-sulfamethoxazole (70.4%), and tetracycline (62.8%) (45). A review by Tadesse et al. on AMR in humans found that a high level of drug resistance existed to commonly prescribed antibiotics in the African continent (46). *E. coli* was commonly isolated from patients with bloodstream infections (17/87, 19.5%), urinary tract infections (17/87, 19.5%), and wound infections (16/87, 18.4%) (46). Resistance to amoxicillin, ampicillin and trimethoprim/sulfamethoxazole was high. Median resistance of *Escherichia coli* to amoxicillin, ampicillin trimethoprim and gentamicin was 88.1, 86.7, and 80.7%, respectively (46). Resistance to either ceftriaxone or cefotaxime, which is suggestive of extended–spectrum beta lactamase (ESBL) production was reported in 20.0% of *E. coli* (46).

This study also found tetracycline and sulfamethoxazole/trimethoprim, to be among the most commonly used antibiotics for treatment, among the poultry farmers interviewed, during the production process. However, ampicillin was not among the commonly used antibiotics during the production process. A study from Tanzania further, indicated that oxytetracycline and sulphadimidines were the most used antibiotics and that few broiler farmers used antibiotics for growth promotion (43). Supplements given to poultry during production contain antibiotics such as oxytetracycline, doxycycline, and sulfamethoxazole, to which the birds were constantly exposed; while other antibiotics like amoxicillin, oxytetracycline, and ceftriaxone were excessively used for the treatment of infections during the production process (44). Over-the-counter-sale of some antibiotics without prescriptions (47) and inadequate AMR surveillance in food animals could exacerbate the problem of AMR.

Studies conducted in Zambia, including Lusaka Province on AMR in broiler chickens, showed *E. coli* resistance to tetracycline, ampicillin, and sulfamethoxazole/trimethoprim (11, 12). Phiri et al. observed the prevalence of AMR to be 94.6% and resistance to tetracycline (81.4%), sulfamethoxazole/trimethoprim (65.4%), and ampicillin (72.9%) among *E. coli* isolates from broilers at abattoirs and open markets (11). Similarly, Muligisa-Muonga et al. found prevalence of AMR to be 88% and resistance to tetracycline (79.4%), sulfamethoxazole/trimethoprim (49.7%), and ampicillin (51.9%) of *E. coli* isolates from retail broiler carcasses (12).

Generally, an increase in the prevalence of ESBL-producing *E. coli* in Africa and around the world has been observed (48, 49).

In terms of genetic determinants of AMR, TEM and SHV genes have been frequently reported. However, recently, *CTX-M* has been the most implicated in both humans and broilers (40, 41).

Of the targeted ESBL-producing resistance genes for this study, *CTX-M* was the only gene detected, similar to what was found by Dahms et al. (50). The other two targeted genes, *TEM* and *SHV*, were not detected despite the phenotypic data showing ESBL production. This disparity could be due to differences in the sequences of the target genes. The detection of *CTX-M* could be attributed to the fact that *CTX-M* producing ESBLs have become more prevalent in the recent past (51, 52) as reported in the review done by Kawamura et al. (53).

The detection of ESBL-producing strains of *E. coli* is of great concern to the human, food animal populations and their environments, as these microorganisms can easily be transferred or acquired through excessive bacterial growth and cross-contamination as a result of poor food handling, consumption of contaminated food or poor sanitation. A study by Hedman et al. revealed high *CTX* resistance (66.1%) in farmed broiler chickens, an increase in *CTX* resistance over time in backyard chickens not fed antibiotics (2.3–17.9%), and identical *bla*__*CTX*_−M_ sequences from human and chicken bacteria, suggesting a spillover event (35). Another study conducted in the Netherlands showed a genetic similarity among the ESBL producing *E. coli* strains found in retail chicken meat and those in healthy but infected humans (54). AMR in humans is interlinked with AMR in other populations, especially farm animals and in the wider environment (38).

AMR leads to early empirical treatment failure, limitations in the choice of antibiotics, and possible complications resulting from the failed treatment (27, 28). Zambia has experienced the negative effects of resistant pathogenic microorganisms as evident in a study by Songe et al. (25) who demonstrated resistance to some foodborne pathogens (non-typhoidal Salmonellae and *Shigella flexneri* and S. *dysente*ries) in patients with HIV-related persistent diarrhea. In addition, a review by Mshana et al. (55) showed an increasing trend in the incidence of antibiotic resistance in Zambia, the Democratic Republic of the Congo, Mozambique, and Tanzania. Of significant concern has been the increase in multidrug-resistant *Escherichia coli, Klebsiella pneumoniae, Staphylococcus aureus, Vibrio cholerae*, non-typhoid *Salmonella*, and other pathogens responsible for nosocomial infections.

Zambia has a challenge of high poverty levels (>60%) (56). Poverty and malnutrition are endemic in rural areas (56). One of the major causes is the country's high disease burden (57) due to food safety issues among others. The sick and caregivers do not contribute effectively to the country's economy, and this worsens poverty. It is, therefore, a cause for concern wherever antimicrobials are in use.

## 5. Conclusion and recommendations

Most of the broiler farmers were aware of antibiotic resistance though they were not aware of the impact of antibiotic resistance on human health. Through the “One Health” approach, the government should increase awareness on AMR in humans, food animal production, the environment and its possible public health threat. Awareness among food animal farmers and local consumers is important considering their strategic position in the food chain.

The prevalence of AMR *E. coli* was relatively high, with the highest resistance to tetracycline, sulfamethoxazole/trimethoprim, and ampicillin which could be attributed to the use of antibiotics in humans for treatment of infections, food animal production and the spread of AMR microorganisms. More research is needed to identify the subgroups of *E. coli* and further molecular research to determine the genes that confer this resistance against antibiotics. In addition, more studies should also be conducted to identify the sources and administration of antibiotics particularly among small scale broiler farmers.

There is a need to regulate the use of and access to antibiotics through strict legislation for pharmacies, pharmaceutical companies as well as agro-vet shops. Stewardship programs are essential and need to be developed and supported to ensure that human and veterinary antibiotics are used properly.

The government should develop and maintain registers of food animal farmers such as poultry farmers. There is also a need for surveillance of AMR; in human, poultry and other livestock and the environment, for assessing the spread of AMR and inform policies.

The study was limited in scope and studies to look at poultry meat, live birds, and the environment are needed along with human data.

## 6. Study limitations

Statistically significant findings that are really present in a study population are hard to detect with small sample size. The small number of samples against the computed number was that we could only manage to locate a small number of farmers that were in production. The lack of a database contributed to the challenge of locating the broiler farmers to include in our study and hence failed to meet the target sample size. Additionally, some broiler farmers reared their chickens in spare rooms within their main houses, making it difficult to be identified in the neighborhood. Further, the use of a semi-structured questionnaire had potential limitations such as recall bias and interpretation issues; however, it still gave some insight into the AMR problem among the broiler poultry farmers in Lusaka Province.

## Data availability statement

The data presented in the study were deposited in National Center for Biotechnology Information (NCBI) accession numbers are as follows: SUB12488218 ks13 OQ121818, SUB12488218 LS28 OQ121819, SUB12488218 LS281 OQ121820, SUB12488218 LS171 OQ121821, SUB12488218 CS02 OQ121822, SUB12488218 CS13 OQ121823.

## Ethics statement

The studies involving human participants were reviewed and approved by ERES Converge Ethics Committee (IBR No. 00005948). Affiliation is non-governmental. The patients/participants provided their written informed consent to participate in this study.

## Author contributions

JM, GM, MMu, MMw, and GK conceived and designed the study idea. MMu, MMw, EM, and IS were involved in data collection. MMw, GK, NP, EM-M, and KY analyzed the laboratory samples. MMu, MMw, EM, GK, and JM were involved in the interpretation of the results. MMu and JM drafted the manuscript. GM, GK, and FB reviewed and edited the manuscript. JM coordinated the study. All authors contributed to the article and approved the submitted version.
